# Enhanced Expression of Secreted α-Klotho in the Hippocampus Alters Nesting Behavior and Memory Formation in Mice

**DOI:** 10.3389/fncel.2019.00133

**Published:** 2019-04-02

**Authors:** Dongxue Li, Dongqing Jing, Ziyang Liu, Ying Chen, Fang Huang, Thomas Behnisch

**Affiliations:** Institutes of Brain Science, State Key Laboratory of Medical Neurobiology and Collaborative Innovation Center for Brain Science, Fudan University, Shanghai, China

**Keywords:** klotho, object recognition and location memory, hippocampal formation/hippocampus, passive avoidance memory, anti-aging, nesting behavior

## Abstract

The klotho gene family consists of α-, β-, and γ-Klotho, which encode type I single-pass transmembrane proteins with large extracellular domains. α-Klotho exists as a full-length membrane-bound and as a soluble form after cleavage of the extracellular domain. Due to gene splicing, a short extracellular Klotho form can be expressed and secreted. Inactivation of α-Klotho leads to a phenotype that resembles accelerated aging, as the expression level of the α-Klotho protein in the hippocampal formation of mice decreases with age. Here, we show that intrahippocampal viral expression of secreted human α-Klotho alters social behavior and memory formation. Interestingly, overexpression of secreted human α-Klotho in the CA1 changed the nest-building behavior and improved object recognition, object location and passive avoidance memory. Moreover, α-Klotho overexpression increased hippocampal synaptic transmission in response to standardized stimulation strengths, altered paired-pulse facilitation of synaptic transmission, and enhanced activity-dependent synaptic plasticity. These results indicate that memory formation benefits from an augmented level of secreted α-Klotho.

## Key Points

- The putative anti-aging α-Klotho protein exists as a full-length membrane-bound, as a soluble form and due to gene splicing, as a short extracellular Klotho form – the secreted α-Klotho.
- We examined the effects of secreted human α-Klotho overexpressed in the excitatory CA1 neurons of the dorsal-intermediate hippocampus on nesting behavior, object recognition, location memory and fear memory in 11- to 12-month-old mice.
- Secreted α-Klotho overexpression enhanced memory performance in all tasks and altered significantly the nest building behavior.
- Secreted α-Klotho overexpression reduced the expression level of synaptic vesicle protein SNAP 25 and attenuated paired pulse facilitation of glutamatergic neurotransmission, but increased evoked excitatory transmission.
- Secreted α-Klotho overexpression altered activity dependent synaptic potentiation, but not synaptic depression.
- These results indicate that memory formation benefits from an augmented level of secreted α-Klotho but that some aspects of social behavior were altered.

## Introduction

The klotho gene family consists of α-, β-, and γ-Klotho, which encode type I single-pass transmembrane proteins that were originally described as co-receptors for endocrine fibroblast growth factors (Kuro-o et al., 1997; Ito et al., 2000; Kurosu et al., 2007; Fon Tacer et al., 2010). α-Klotho is a pleiotropic protein that has been found to delay aging and to enhance cognition in mice (Nagai et al., 2003; Kurosu et al., 2005; Dubal et al., 2014). α-Klotho encodes a type I transmembrane protein consisting of 1014 amino acid residues in mice and rats (Kuro-o et al., 1997; Shiraki-Iida et al., 1998) and 1012 amino acid residues in humans (Matsumura et al., 1998). Due to gene splicing, there are two transcripts encoding a full-length protein with intracellular, transmembrane and extracellular domains and a protein that only consists of a part of the extracellular domain, the so called secreted α-Klotho protein. Since the secreted α-Klotho protein does not require cleavage, it can be secreted directly into the extracellular space, reaching the cerebrospinal fluid and blood serum (Matsumura et al., 1998). The proteolytic shedding of Klotho takes place not only in cells of the kidney but also in those of the brain (Chen et al., 2014). The full-length extracellular domain can also be released into the serum and CSF after cleavage by a disintegrin and metalloproteinase domain-containing proteins (ADAMs), allowing it to circulate throughout the body (Saftig and Lichtenthaler, 2015; Peron et al., 2018). ADAM10 and ADAM17 have α-secretase activity that is also responsible for the cleavage of the amyloid precursor protein (APP).

α-Klotho is expressed by the choroid plexus in the lateral ventricle; however, its transcripts have been detected in a variety of neuronal cell types throughout the brain, including in the hippocampus (Clinton et al., 2013). In particular, the secreted form might suppress oxidative stress and growth factor signaling and regulate ion channels and transporters. In particular, its over-expression seems to be able to suppress insulin/IGF-1 signaling and to extend life span (Sopjani et al., 2015).

It has been observed that the brain level of Klotho decreases with aging in monkeys (Duce et al., 2008). Klotho levels in the CSF of humans also decrease with aging and in AD (Semba et al., 2014). Genetic knockdown of Klotho in mice reduces life span (Kuro-o et al., 1997) and impairs myelination (Chen et al., 2013; Chen et al., 2015), synaptic integrity (Shiozaki et al., 2008), and cognition (Nagai et al., 2003). In contrast, overexpression of Klotho protects neuronal cultures against amyloid beta oligomers (Zeldich et al., 2014). In humans, a genetic variant of Klotho, KL-VS, increases the circulating levels of Klotho and promotes longevity (Arking et al., 2002; Invidia et al., 2010) and cognitive functions (Dubal et al., 2014) in normal aging populations. However, it is less clear whether α-Klotho elevation can counteract cognitive disorders, such as Alzheimer’s disease. Since Klotho is releasable into the blood stream and can act as a hormone on multiple cellular targets simultaneously, Klotho has the potential to be used in gene therapy to attenuate memory decline. Here, we utilized a CRE-double-floxed expression system to overexpress human secreted α-Klotho in hippocampal CA1 neurons of aged mice and to study the effects of overexpressed α-Klotho on object recognition and object location memory (OLM) and synaptic transmission.

## Materials and Methods

### Ethics Statement

Efforts were made to minimize the number of animals sacrificed. This study was carried out in accordance with the recommendations of the Institutes of Brain Science and State Key Laboratory of Medical Neurobiology of Fudan University, Shanghai, China and approved by the Institutional Animal Care and Use Committee of Fudan University, Shanghai Medical College (IACUC Animal Project Number: 31320103906). The protocol was approved by the Institutes of Brain Science, Fudan University, Shanghai, China.

### Animals

Eleven -twelve -month-old CaMKIIalpha-cre T29-1 +/+ transgenic male mice (Jackson lab: T29-1; 005359, B6.Cg-Tg(Camk2a-cre)T29-1Stl/J) were housed with a 12 h reverse dark/light cycle at 23°C and free access to food and water. Litter maters were studied. The outline of the experimental sequence has been depicted in the Supplementary Figure 1.

### AAV Serotype 9 and α-Klotho

The sequence (Addgene ID: 17713) of secreted human α-Klotho was subcloned into an Adeno-Associated Virus serotype-9 vector encoding a CAG promoter, double flox, 2A and GFP (AAV9-CAG-FLEX-KL-2A-GFP) using standard cloning techniques. The multi cloning site was in between the double floxes. The sequence of human secreted Klotho was subcloned into pAAV9 vector after PCR amplification. Recombinant AAV9 was produced and tested in cell cultures (HeLa) and reached 5 × 10^12^ vg/mL. The AAV9s were stored at -80°C after aliquoting into 5 μl volumes. To create control plasmid, the same subcloning and AAV9 production steps were followed with the AAV9-CAG-FLEX-2A-GFP plasmid only.

The change in body weight of the Klotho-2A-GFP and GFP-only transduced mice remained similar. In comparison to the body weight at the day of transduction, the weight of the mice overexpressing Klotho increased to 26.7 ± 0.46 g and the weight of GFP-only mice to 26.0 ± 0.29 g.

### Stereotaxic Intra-Hippocampal Injections of AAV9

The mice were anesthetized with isoflurane (approximately 4%, Veteasy, RWD Life Science) to reach a level of anesthesia without corneal reflexes, pedal reflexes, twitches of the large facial whiskers and tail reflexes. The mice were then secured in a stereotaxic frame (Stoelting, Wood Dale, IL, United States). The craniotomy procedure followed closely the protocol that was published by Cetin et al. (2006). Glass pipettes (Harvard Apparatus, Holliston, MA, United States) with a tip length of about 10 mm and a tip diameter of about 30 μm were filled with a buffer solution containing the AAV9. At the respective coordinates, a hole of 0.5 mm diameter was drilled in the skull through which the glass pipette was lowered down for about 3 mm to reach the stratum pyramidale of the CA1 hippocampal region. The anterior-posterior (AP) coordinate was -3.5 mm and the lateral (L) position was ± 3.5 mm. The stereotaxic coordinates were chosen to transduce hippocampal CA1 neurons within the dorsal and intermediate part of the hippocampus along its septotemporal axis (Fanselow and Dong, 2010; Wang et al., 2017). The injection was controlled by a Hamilton syringe (CR-700-50, Hamilton Co., Hoechst, Germany). The vehicle or AAV9 (1.5 μL) were injected at three depths into both hippocampi. At every depth 0.5 μL of AAV9 were injected (Masso et al., 2017; Raza et al., 2017; Wang et al., 2017).

For the post-surgical care, a cream (Neosporin, Johnson & Johnson, United States) that contained two antibiotics (Neomycin, Polymyxin B) and the analgesic Pramoxine hydrochloride has been applied topically after surgery to the incision site. The treatment was continued four times per day for a total of 3 days. The animals were kept warm with a heat lamp for at least 24 h after surgery. The behavior of the animals was closely monitored for a week to make sure that they reached wetted food and water.

**FIGURE 1:**
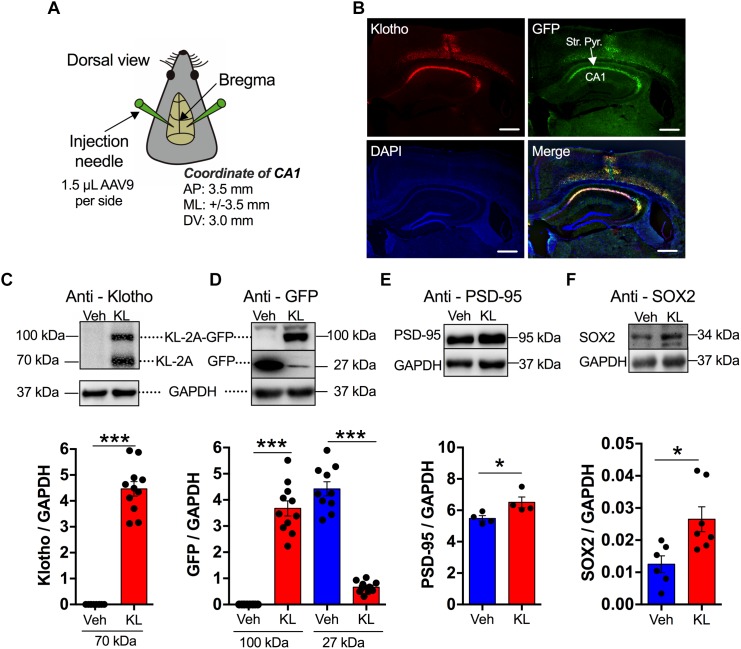
Overexpression of human secreted α-Klotho in hippocampal CA1 neurons. **(A)** The schematic diagram shows the stereotaxic coordinates of the intra-hippocampal injection sites of the AAV9 titre (AP: anterior-posterior; ML: mediolateral; DV: dorsal-ventral). **(B)** Representative fluorescence (GFP, green; DAPI, blue) and immunofluorescence (anti-α-Klotho, red) images. The anti-α-Klotho immunofluorescence (red) is co-localized with GFP fluorescence (green) in the CA1 region. The nuclear marker DAPI indicates the cell layers (blue). Horizontal scale bar: 500 μm. **(C–F)** The inserts show representative Western blots of Klotho (*n* = 11) vs. Vehicle (*n* = 10), GFP (*n* = 11) vs. Vehicle (*n* = 10), PSD-95 (*n* = 4) vs. Vehicle (*n* = 4) and SOX2 (*n* = 7) vs. Vehicle (*n* = 6) in the hippocampal formation with their corresponding GAPDH blots. The significant increases in α-Klotho, PSD-95, and SOX2 levels in the Klotho group compared to the Vehicle groups are summarized in the bar graphs. Asterisks and horizontal lines: *t*-test: ^∗^*P* < 0.05; ^∗∗^*P* < 0.01.

The overexpressed Klotho or GFP levels were verified for all animals studied using either Western blotting of whole hippocampus lysate against Klotho (Figure 1C,D: Abcam 181373, and GFP (Beijing Biodragon Immunotechnologies, B1025) or by fluorescence imaging. The localization and expression level were analyzed using wide-field fluorescence imaging, in case of electrophysiological experiments.

### Western Blotting

The animals were anesthetized and transcardially perfused briefly with 50 ml ice-cold 0.9% saline and then the brain isolated and trimmed for Western blotting analysis (Wang et al., 2017; Yun et al., 2018). The hippocampi were isolated mechanically by needles (Shetty et al., 2015). The isolated hippocampi were transferred into eppendorf tubes and immediately immerged into liquid nitrogen for the snap-freezing. Whole cell proteins were extracted from the hippocampus using RIPA lysis buffer (Beyotime: Tris (pH 7.4), 150 mM NaCl, 1% Triton X-100, 1% sodium deoxycholate, 0.1% SDS and sodium orthovanadate, sodium fluoride, EDTA, leupeptin) with protease inhibitor (Roche: 04693124001) and phosphatase inhibitor (Roche: 04906837001). After ultra-sonication, the samples were centrifuged (4°C, 12000 rpm, 5 min) and the supernatant was removed from the pellet, mixed with 4 × SDS loading buffer, boiled for 8 min and then stored at -80^°^C. Protein concentrations were determined by the bicinchoninic acid method (BCA Protein Assay Kit, Beyotime: P0012S, China). Equal amounts of proteins were separated on an SDS gradient gel and then transferred onto polyvinylidene difluoride membranes (Millipore: IPVH00010). After blocking in 5% fat-free milk for 1 h, the membranes were incubated with primary antibodies overnight at 4°C. On the next day, the membranes were washed and incubated with secondary antibodies. The blots were then incubated with the chemofluorescent reagent ECL (Thermo Scientific, Rockford, IL, United States). Antibodies and suppliers of primary antibodies: rabbit anti-Klotho: (1:1000; Abcam: 154163, 181373); mouse anti-GFP: (1:5000, Beijing Biodragon Immunotechnologies, B1025); rabbit anti-p-Akt (Ser473): (1:2000; Cell Signaling Technology: 4060); rabbit anti-Akt: (1:1000; Cell Signaling Technology: 4685); rabbit anti-GAPDH: (1:1000; Cell Signaling Technology: 2118); rabbit anti-SOX2: (1:1000; Abcam: 97959); rabbit anti-PSD-95: (1:1000; Cell Signaling Technology: 2507); anti-p-PI3K: (1:1000; Cell Signaling Technology: 4228T); anti-PI3K: (1:1000; Cell Signaling Technology: 4257T); rabbit anti-β-tubulin: (1:1000; Cell Signaling Technology: 2146); rabbit anti-Snap 25: (1:1000; Cell Signaling Technology: 5309); rabbit anti-Munc18-1: (1:1000; Cell Signaling Technology: 13414); rabbit anti-Syntaxin 1A: (1:1000; Cell Signaling Technology: 13002). Secondary antibodies: horseradish peroxidase-conjugated goat anti-rabbit: (1:10000; Jackson ImmunoResearch West Grove: 111-035-003); horseradish peroxidase-conjugated goat anti-mouse: (1:10000; Jackson ImmunoResearch West Grove: 115-035-003).

### Immunofluorescence

The animals were anesthetized with isoflurane (4% isoflurane) and transcardially perfused briefly with 50 mL ice-cold 0.9% saline and after decapitation the brain was isolated (Wang et al., 2017). The hemispheres were kept overnight in 4% paraformaldehyde. The fixed brains were kept for at least 3 days in 30% saccharose solution in PBS. Sections (30 μm) were prepared using a Cryotome (Leica, Germany). Sections were placed and permeabilized with 0.3% Triton X-100 in PBS 30 min and non-specific binding-sites were blocked with 5% goat serum in PBS for 2 h at room temperature and incubated overnight at 4°C with primary antibody (anti-Klotho, 1:500; Abcam 154163). After three times washing in PBS, sections were incubated for 1 h with secondary species-specific antibody (goat-anti-rabbit Alexa 546, 1:400; Invitrogen) in 5% goat serum. After 5 min of DAPI in 0.01 M PBS (100 ng/mL, 28718-90-3, Roche, Switzerland) administration and three times washing in PBS the sections were mounted (Fluoromount Aqueous Mounting Medium, Sigma, United States) on slides and fluorescence images acquired with a fluorescent microscope system. The GFP fluorescence was detected directly without antibody staining. Representative fluorescence image of transduced CA1 neurons and bar diagrams of the Integral density/area of fluorescence structures of electrophysiological experiments are shown in Supplementary Figure 3.

### Behavioral Experiments

#### Observation of Nest Building Behavior

Sixteen mice per group have been split equally into 4 cages for evaluation of the nest building behavior. Every cage represents a batch of 4 animals that were infected at the same day. Klotho and Vehicle injections took place at the same day (4 + 4 mice). Thirty cotton balls (about 1 cm diameter) were placed in the center of the cage floor housing four mice. The degree of biting cotton balls into pieces and their collection in a corner were analyzed at different time points within a 15-cell grid (Figure 3A). Values are presented in Supplementary Table 2.

#### Passive Avoidance Task

The equipment consists of a transparent box with light (19 cm × 17cm × 21 cm) and an interconnected box without light (dark box, 20 cm × 17 cm × 21 cm). The floor of the dark box contains strings of inter-isolated steel tubes, through which an electric current with opposing polarity was applied, allowing the application of brief foot shocks. The two chambers are connected through a guillotine shape-like door. The training session began with a 30 s period of habituation, during which the inter-compartment door had been kept closed. After the initial 30 s period, the door opened automatically. When the animal crossed from the light compartment to the dark one, the mouse received a 0.3 mA food shock for 2 s period. To avoid early escape and to assure a 2 s stimulation time, the inter-compartment door were kept closed once a mouse had entered the dark box. The degree of memory retention has been evaluated by measurement of the time an animal required to enter the dark box (crossing latency) at different time points (Figure 4A) (Wang et al., 2017). The mice were allowed to enter the dark box only once in all trials. If an animal did not enter into the dark box within 300 s, then this mouse was placed back into the housing cage and the crossing latency was recoded as 300 s. A certain mouse was trained and tested always in the same box to avoid novelty. Four passive avoidance systems were available. The floor and the wall of the system for the passive avoidance task was cleaned with 70% ethanol to minimize olfactory cues after each mouse was trained or tested.

#### Object Location and Recognition Memory

To habituate mice for the object recognition memory (ORM) task, the animals were allowed to explore a round black barrel (diameter: 26 cm and height 38 cm) for 30 min per day at three consecutive days and for an additional 10 min at the fourth day. On the fifth day, the animals were trained to memorize two objects (20 cm distance) within 6 min. After 24 h retention interval, a novel object with a different shape and color was taken to replace one of the objects. The exploration time for the novel and the familiar objects was measured over a 6 min interval and the values used to calculate the discrimination index (DI). The DI was calculated as follows (Vogel-Ciernia and Wood, 2014; Wang et al., 2017): (time exploring the novel object – time exploring the familiar object)/(time exploring novel object + time exploring familiar object) ^∗^ 100%.

After a rest for 5 days, the same animals underwent an OLM task. To this end, the animals were habituated to an empty white box (30 cm × 26 cm × 30 cm) for 30 min per day for three consecutive days and 10 min at the fourth day. During the training session at the fifth day, the mice freely explored the floor of the box that contained two different objects. To get a measure of the OLM, one object was moved 24 cm to a new position and the total time of exploration of the familiar and novel object localization was measured 24 h after the training (Vogel-Ciernia and Wood, 2014). The objects and boxes were cleaned with ethanol after every training or test. An outline of the procedure is depicted in Figure 5A.

### Electrophysiology

#### Preparation of Acute Hippocampal Slices

The preparation of acute hippocampal slices followed previously published methodologies (Weng et al., 2018; Yun et al., 2018). Briefly, after anaesthetization with isoflurane inhalation (4%) and decapitation, brains were immersed in ice-cold ACSF (95% O2/5% CO2 and a composition in mM: 124 NaCl, 2.5 KCl, 2.5 CaCl2, 2 MgCl2⋅6H2O, 1.25 KH2PO4, 10 glucose, 26 NaHCO3, pH 7.3–7.4). Immediately after preparation of the slices using a vibratome (VT-1200, Leica, Germany). These transverse hippocampal slices (350 μm) were incubated in a submersion-incubation chamber at 30°C for at least 2 h. Electrophysiological experiments were performed in a submerged type recording chamber system (Warner Instruments, RC-26GLP) mounted on a Nikon Eclipse FN1 microscope with constant perfusion (4 mL/min) of ACSF at 30°C. In the electrophysiological experiments, “n” states the number of slices. In addition, the number of “n” in the LTP and LTD experiments corresponds to the number of animals. For the PPF, I/O characterization experiments not more than two slices from one animal were analyzed.

#### Recording of Field Excitatory Postsynaptic Potentials

Field excitatory postsynaptic potentials (fEPSPs) were recorded in the stratum radiatum of the hippocampal CA1 area via borosilicate micropipettes filled with ACSF (Weng et al., 2016; Yun et al., 2018) in a submerged slice chamber mounted to a Nikon Fluorescence microscope equipped with a PCO.Edge video camera. Therefore, the expression of GFP in acute hippocampal slice was monitored and analyzed. Biphasic stimulation currents (100 μs/pulse width) were applied through monopolar metal stimulation electrode (575500 tungsten electrodes, A-M Systems, United States) to stimulate Schaffer collateral fibers every minute. Recorded evoked field potentials were amplified by MultiClamp 700B (Axon, United States) and filtered (2.8 kHz low-pass, 0.1 Hz high-pass) and then digitized at a sample frequency of 20 kHz (Digidata 1440A, Clampex 10.2, Molecular Devices, United States). Stimulation strengths were adjusted to 30–45% of the maximum of fEPSP slope values. fEPSP slopes or fEPSP amplitudes were determined and expressed as a percentage of baseline values.

### Statistical Analysis

Data are expressed as mean ± standard error of mean (SEM.). The values of different experimental conditions were compared using *t*-Test (Western blots), however, when the Shapiro-Wilk test for normality was significant then the Mann-Whitney *U* test was applied. In addition, the group effect on repeatedly measured behavioral parameters were tested using Repeated Measurement ANOVA [SPSS (United States)]. Non-parametric tests were chosen because a normal distribution (Shapiro-Wilk test) could not always be assumed (Sajikumar et al., 2007; Salkovic-Petrisic et al., 2015; Panday and Rauniar, 2016). *P*-value with <0.05 was considered to indicate a statistically significant difference between two groups and presented in the text. All tests were analyzed two-tailed. The corresponding *F*-, *t*- or *Z*-values and degree of freedom (df) for Fisher’s test, t-Test and Mann-Whitney U test, respectively, are documented in Supplementary Tables 1–7. The capital letter “N” indicates the number of batches analyzed for the Nesting behavior and “n” indicates the number of individual analyses. Klotho overexpression experiments were interleaved with GFP-only expression studies (Vehicle).

## Results

### Viral Expression of Human Secreted α-Klotho in Hippocampal CA1 Neurons

To learn more about the age-dependent variation of endogenous α-Klotho expression in the hippocampal formation, the CA1, CA3 and DG area were analyzed separately using Western blotting. The data indicated that the expression level within these areas correlated negatively with age. The results for the one-way ANOVA revealed significant effects for age on the Klotho-expression level within the DG [*F*(4,10) = 17.82, *P* = 0.0002], the CA3 [*F*(4,10) = 51.13, *P* < 0.0001], and the CA1 [*F*(4,10) = 49.29, *P* < 0.0001]. The highest Klotho level was detected at the age of 3-month in DG and CA3 and 1-month in the CA1 area. The lowest level were observed at the age of 16-month in the DG (Supplementary Figures 2A,B). Thus, the level of endogenous Klotho is age-dependently regulated.

We first conducted a study to counteract the decline in α-Klotho expression through artificial overexpression of the human-derived α-Klotho sequence in CA1 neurons utilizing an AAV9-based expression system in 11-month-old mice. As presented in Figure 1, stereotaxic intra-hippocampal infection of the CA1 neurons led to a detectable and region-specific overexpression of α-Klotho in the dorsal-intermediate area of the hippocampus (Figure 1A,B). The acute hippocampal slices for electrophysiological experiments were utilized to verify the expression level of Klotho or Vehicle as it has been summarized in the Supplementary Figure 3. The expression level was estimated by the GFP based fluorescence signal in the stratum pyramidale and normalized to the integrated area. The data show a specific labeling of neurons in the CA1 area.

Enhanced Klotho expression was also verified by Western blotting or immunofluorescence after behavior tests or electrophysiological recordings (Figure 1C,D and Supplementary Figure 4). The Western blots indicated α-Klotho levels at 70 kDa (*n* = 11) of approximately 4 in comparison with approximately 0 in the Vehicle group (Vehicle: *n* = 10; Klotho vs. Vehicle: *t* = -15.083, df = 19, *P* < 0.001, Figure 1C,D). The human-derived sequence of α-Klotho consists of the nucleotide sequence of the extracellular Klotho domain 1 (KL1). Thus, the anti-α-Klotho immunosignal corresponds to approximately 62 kDa, however, a second immunosignal at 100 kDa was observed. This 100 kDa immunosignal corresponded to a protein that consists of secreted α-Klotho protein (approximately 62 kDa) and GFP (approximately 27 kDa). The 100 kDa band was positive for GFP. This indicated that the efficiency of the expression of the unattached vector by the 2A system was low but was sufficient to increase the level of GFP-detached secreted α-Klotho. Under all experimental conditions, animals with GFP-only expression were used as the Vehicle group. Both the Vehicle group (immunosignal: 4.42 ± 0.271, *n* = 10) and the Klotho group (immunosignal: 0.665 ± 0.064, *n* = 11; Klotho vs. Vehicle: *t* = 14.090, df = 19, *P* < 0.001) had GFP expression at the 27 kDa position, but only the Klotho group showed a GFP-positive immunosignal at the approximately 100 kDa position (immunosignal: 3.67 ± 0.287, *n* = 11; Klotho vs. Vehicle: *t* = -12.197, df = 19, *P* < 0.001; Figure 1D). To evaluate possible modifications in synaptogenesis in the hippocampal formation, the expression level of the postsynaptic density protein PSD-95 was evaluated. The data showed that a 2-week overexpression of secreted Klotho significantly increased the amount of PSD-95, indicating that some modulation of the scaffolding protein network of synapses occurred. The PSD-95 level was 6.5 ± 0.34 in Klotho overexpressing mice and 5.5 ± 0.18 in the Vehicle mice (*t*-test: *P* = 0.037, *n* = 4 for each group, Figure 1E). Since soluble α-Klotho (after cleavage from full-length α-Klotho) has been shown to promote neurogenesis (Collins, 2018; Fernandez et al., 2018), the level of neurogenesis was characterized utilizing SOX2 as a marker. The expression level of SOX2 was 0.03 ± 0.0043 (*n* = 7) in α-Klotho mice, and significantly greater than that in Vehicle mice with 0.01 ± 0.0026 (*n* = 6; *t*-test: *P* = 0.011, Figure 1F). Details of the statistical comparisons between the groups (*F*-, *t*-values and df values) are presented in Supplementary Table 1.

Thus, the data show the efficient transduction of CA1 neurons. In addition, the data indicate that the overexpression of Klotho alters the molecular composition of synapses and promotes neurogenesis.

### Prolonged Overexpression of Human Secreted Klotho Enhances Key Elements of Synaptic Plasticity Related Signaling Pathways

We were interested in determining whether signaling pathways that have previously been linked to synaptic plasticity were altered. We did not observe (data not shown) significant differences between α-Klotho-overexpressing and Vehicle mice regarding the phosphorylation level of calmodulin-dependent kinase II (CaMKII), 6.5 ± 0.44 (Vehicle, *n* = 6) vs. 6.8 ± 0.32 (Klotho, *n* = 7, *t*-test: *P* = 0.73), the phosphorylation level of the transcription factor CREB 0.02 ± 0.004 (Vehicle, *n* = 6) vs. 0.004 ± 0.015 (Klotho, *n* = 7, *t*-test: *P* = 0.4), or the phosphorylation level of eEF2 0.3 ± 0.01 (Vehicle, *n* = 6) vs. 0.3 ± 0.03 (Klotho, *n* = 7, *t*-test: *P* = 0.69).

**FIGURE 2:**
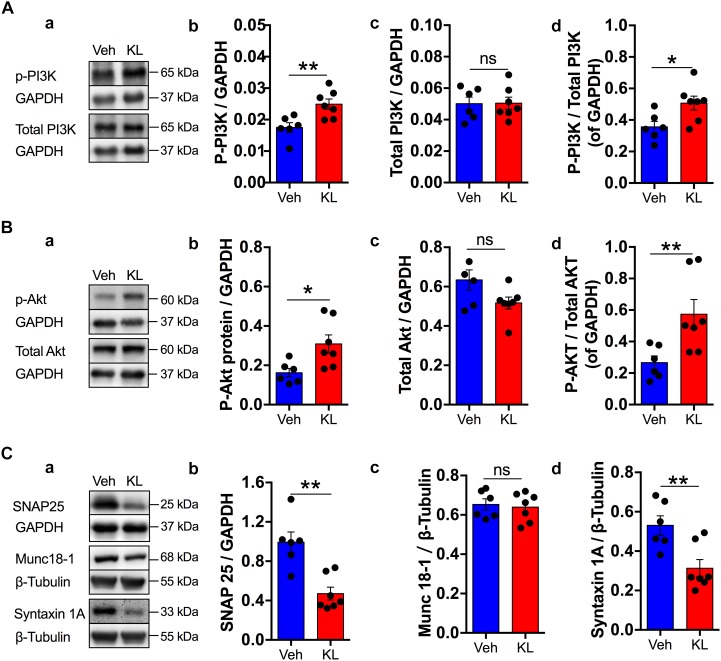
Effects of the overexpression of human secreted Klotho on some synaptic plasticity-related enzymes. The inserts in **A–C** **(a)** show representative Western blots for the indicated proteins with their corresponding GAPDH or beta-tubulin levels for normalization (*n* = 6: Veh: Vehicle; *n* = 7: KL: α-Klotho). Panel **A** summarizes the effects of Klotho overexpression on p-PI3K **(b)**, total-PI3K **(c)** and p-PI3K/total PI3K **(d)** with bar graphs. Panel **B** presents similar bar graphs for p-AKT **(b)**, total AKT **(c)** and p-AKT/total AKT **(d)**. Panel **C** shows the normalized protein expression levels of SNAP25 **(b)**, Munc18-1 **(c)** and Syntaxin 1A **(d)**.The immunosignals of these proteins were normalized to that of beta-tubulin because of their similarity in size to GAPDH. Asterisks and horizontal lines: *t*-test: ^∗^*P* < 0.05; ^∗∗^*P* < 0.01; ns: non-significant.

However, signaling pathways that have been shown to be involved in Klotho-mediated signaling and in synaptic plasticity, such as the PI3K and AKT pathways, differed significantly between the two groups (Vehicle and Klotho, Figure 2A,B). The normalized protein expression of p-PI3K was 0.02 ± 0.0015 in the hippocampal formation in the Vehicle groups (*n* = 6) and 0.02 ± 0.0016 in the Klotho group, with *P* = 0.007 (*t*-test: *n* = 7, Figure 2Ab). However, the total PI3K did not differ between groups (*t*-test: *P* = 0.9, Figure 2Ac). Thus, the ratio of p-PI3K/GAPDH to total PI3K/GAPDH differed significantly between the two groups (*t*-test: *t* = -2.613, df = 11, *P* = 0.024, Figure 2Ad). The p-AKT/GAPDH value was 0.2 ± 0.02 in the Vehicle group (*n* = 6) and 0.3 ± 0.05 in the Klotho group, with *P* = 0.022 (*n* = 7, Figure 2Bb). Additionally, in these cases, the expression levels of the total proteins within the groups were similar and were not significantly different. However, the ratio of p-AKT/GAPDH to total AKT/GAPDH differed significantly between the two groups (*t*-test: *t* = -2.973, df = 11, *P* = 0.013, Figure 2Bd).

A protein that is involved in synaptic vesicle release is SNAP-25. SNAP-25 protein expression was significantly different between the Vehicle and Klotho groups, which had normalized expression levels of 1.0 ± 0.10 (*n* = 6) and 0.5 ± 0.07 (*n* = 7), respectively (*P* = 0.001, Figure 2Cb). The expression level of Munc 18-1 was 0.65 ± 0.028 in the Vehicle group (*n* = 6) vs. 0.64 ± 0.028 in the Klotho group (*n* = 7, *t*-test: *P* = 0.743, Figure 2Cc).

The normalized expression of the vesicle docking protein Syntaxin 1A was 0.53 ± 0.049 in the Vehicle group (*n* = 6), differing significantly from the 0.31 ± 0.044 in Klotho group (*n* = 7, *t*-test: *t* = 3.33, df = 11, *P* = 0.007, Figure 2Cd). Thus, α-Klotho overexpression significantly reduced the expression levels of these presynaptic vesicle marker proteins. Further details of the statistical comparisons of Western blot data between the groups are presented in Supplementary Table 1. In summary, the data show a specific alteration of the molecular elements of vesicle release machinery.

### Altered Nest-Building Behavior of Mice Overexpressing Human Secreted Klotho

**FIGURE 3:**
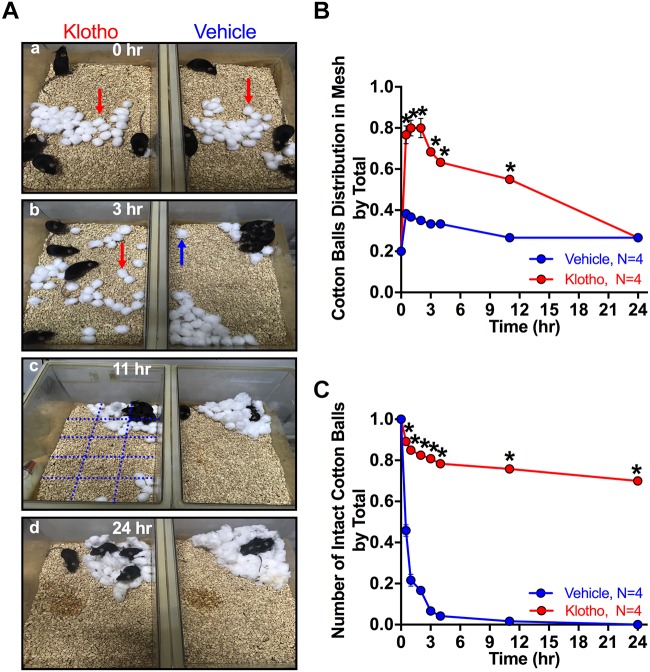
Altered nest-building behavior in human secreted α-Klotho-overexpressing mice. **(A)** Single frame of a video sequence are shown for different times and groups. At time point zero **(a)**, 30 cotton balls were placed in the center of the cage. The degree of cotton ball transfer and shredding was assessed at **(b)** 3 h; **(c)** 11 h and **(d)** 24 h in 4 groups of 4 mice per Klotho group and in four groups of 4 mice per Vehicle group. A grid with 15 squares (black lines in **Ac**) was used to count the number of squares with one or more cotton balls. Red arrow: intact cotton ball; blue arrow: shredded cotton ball; blue grid: used to divide the cage area into 15 squares. **(B)** The human secreted α-Klotho-overexpressing mice distributed cotton balls randomly in the cage within the first hours (red circle: data points; red line: interpolated data). After 24 h, the cotton ball distribution was not different between the Klotho (red) and Vehicle (blue) groups (Mann-Whitney test: ^∗^*P* < 0.05, mean ± SEM, RM-ANOVA time^∗^group effect: ^∗∗∗^*P* < 0.001). **(C)** The degree of cotton ball shredding remained significantly different between the groups over the whole period of observation (RM-ANOVA time^∗^group effect, ^∗∗∗^*P* < 0.001).

For mice, nests are important for heat conservation, reproduction, and shelter. Nesting behavior can be evaluated by analyzing the time and accuracy of nest building utilizing cotton balls placed in the center of the cage. Typically, mice shred the cotton balls into smaller pieces and then arrange those pieces in a cage corner for a nest (Bult and Lynch, 1996). However, the mice that overexpressed Klotho in the CA1 area showed a striking reluctance to build nests (Figure 3A). They randomly distributed the cotton balls in the cage without shredding them (Figure 3B) and started to collect the balls only after a prolonged time of observation (24 h) into one of the cage corners to build a nest. The ratio of cotton balls per grid square to the total number of balls after 11 h was 0.55 ± 0.017 for the Klotho group and 0.27 ± 0 for the Vehicle group, and the difference between the groups was significant (Mann-Whitney test: *P* = 0.011; 16 mice, 4 cages). However, after 24 h, the cotton ball distribution was not different between the two groups (Mann-Whitney test: 0.27 ± 0, Vehicle; 0.27 ± 0, Klotho, *P* = 1, Figure 3Ad). Even so, the degree of cotton ball shredding differed significantly between the two groups and was reduced in the Klotho group. The degree of shredding was estimated by visual inspection of the cotton balls and by calculation of the ratio of the number of shredded balls to the total number of balls (24 h: 0.7 ± 0.01, Klotho; 0.0 ± 0, Vehicle; *P* = 0.013; Figure 3C). Further details of the statistical comparisons of nesting behavior between the groups are presented in Supplementary Table 2. An altered nesting-behavior has been linked to autism-like behavior (Rhee et al., 2018).

### Overexpression of Human Secreted α-Klotho Improves Passive Avoidance Memory Performance

**FIGURE 4:**
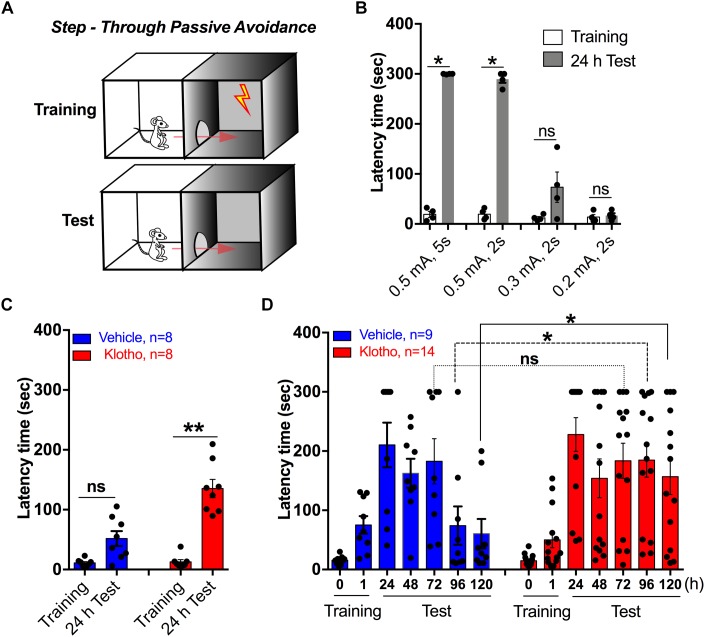
Human secreted α-Klotho overexpression in the hippocampal CA1 region improves short- and long-term passive avoidance memory. **(A)** The schematic diagram shows the sequence of the step-through passive avoidance task. **(B)** The bar graph indicates the effect of different electrical stimulation intensities and durations on step-through latency time (*n* = 4 for each condition). **(C)** One learning trial with 0.3 mA/2 s foot shocks resulted in a moderate increase in step-through latency time in both groups. However, the Klotho group’s latency time was significantly longer than that of the Vehicle group. **(D)** In two-trial training, the Klotho group showed a significant effect in the second training session and at 96 and 120 h. Statistically paired groups are indicated by lines or brackets: Mann-Whitney test: ^∗^*P* < 0.05; ^∗∗^*P* < 0.01. Graphs: black circles: single data points; bars and error bars: mean ± SEM, RM-ANOVA time^∗^group on latency time, ^∗^*P* = 0.033.

Passive avoidance memory requires proper functioning of the hippocampal formation. Thus, the passive avoidance test assesses the degree of retention of a fear memory by measuring the latency time for an animal to enter a dark box where it previously experienced mild electrical foot shocks during training sessions (Figure 4A). The strength of the electrical foot shocks was adjusted to obtain latency times in the 100 sec range in 11- to 12-month-old wild-type mice (0.3 mA/2 s, Figure 4B). This paradigm allowed us to detect a significant memory improvement within 24 h (Mann-Whitney test, *P* = 0.002, Klotho and Vehicle group each *n* = 8, Figure 4C). To enhance the duration of retention, a second set of experiments consisting of two training sessions (Figure 4D) instead of one was utilized (Figure 4C). The degree of avoidance memory was determined 1, 24, 48, 72, 96, and 120 h after the training (Figure 4D). Mice that overexpressed human secreted α-Klotho (*n* = 14) behaved significantly different from the mice of the Vehicle group (*n* = 9). The mice of the Klotho group had a significantly longer latency time than the mice of the Vehicle group 96 h and 120 h after the last training (*P* < 0.05, Figure 4D). For example, 96 h after training, the Klotho group had a latency time of 185 ± 29 s, which was significantly different from the 74 ± 32 s of the Vehicle group (Mann-Whitney test: *P* = 0.017). In addition, the memory retention of the Klotho group was still enhanced compared with that of the Vehicle group 120 h after training (Mann-Whitney test: 157 ± 30 s vs. 61 ± 25 s, respectively; *P* = 0.037; Figure 4D). Details of the statistical comparison of passive avoidance scores between the groups are presented in Supplementary Table 3. The data indicate that enhanced expression of secreted α-Klotho in the hippocampus improves the retention of a learned passive avoidance task while reducing the need to re-establish a fear memory.

### Elevated Expression of Human Secreted α-Klotho Improves Object Recognition Memory (ORM) and Object Location Memory (OLM)

**FIGURE 5:**
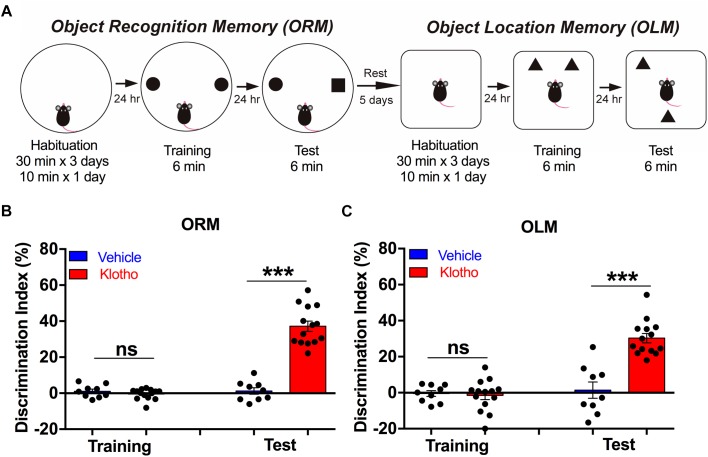
Overexpression of human secreted α-Klotho improves object recognition memory (ORM) and object location memory (OLM). **(A)** The schematic diagram indicates the sequence of the object recognition and object location tasks. **(B)** The bar graph shows that human secreted α-Klotho overexpression significantly increased the ORM, as indicated by the greater discrimination index compared to that of the Vehicle group 24 h after training: Mann-Whitney test: ^∗∗∗^*P* < 0.001). **(C)** The bar graph shows that human secreted α-Klotho overexpression significantly increased the OLM 24 h after training (Mann-Whitney test: ^∗∗∗^*P* < 0.001). Klotho group: *n* = 14; Vehicle group: *n* = 9. Graphs: black circles: data points; bars and error bars: mean ± SEM.

We compared the memory performance of the animals in both groups in an object recognition task followed by an object location task (Figure 5A) by calculating the discrimination index (DI, in %). These tasks allowed us to test whether overexpression of human secreted α-Klotho within the hippocampal CA1 region led to an improvement in memory that was not based on an avoidance reaction but rather on the novelty of objects. While the importance of the hippocampus in object location and ORM is debated, it has been suggested that OLM is mainly hippocampus dependent, whereas ORM involves many other brain regions that could compensate for hippocampal functional declines. Our results showed that in the object recognition tasks, mice overexpressing the secreted α-Klotho had improved memory retention compared with mice of the Vehicle group (Mann-Whitney: Klotho (*n* = 14) 37 ± 3% vs. Vehicle (*n* = 9) 1.2 ± 1.8%; *P* < 0.001; Figure 5B). In the object location task, which was conducted 5 days later, mice overexpressing secreted α-Klotho again showed improved memory retention compared with Vehicle mice (Mann-Whitney: Klotho (*n* = 14) 32 ± 2.9% vs. Vehicle (*n* = 9) 1.4 ± 4.56%, *P* < 0.001; Figure 5C). Details of the statistical comparisons of ORM and OLM scores between the groups are presented in Supplementary Table 4. Thus, enhanced level of Klotho contributes to an improvement of both ORM and OLM.

### Viral Expression of Secreted α-Klotho in the Hippocampal CA1 Region Alters Synaptic Transmission

**FIGURE 6:**
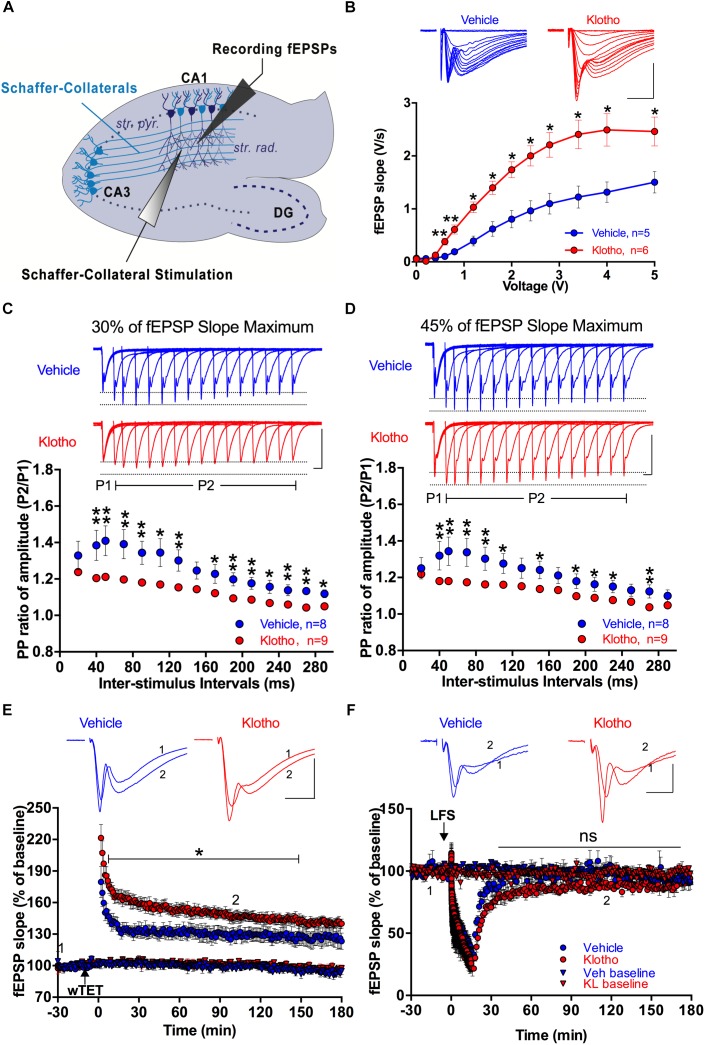
Effects of α-Klotho overexpression on synaptic transmission in acute hippocampal slices. **(A)** The relative positions of Schaffer collateral stimulation and fEPSP recording electrodes are depicted in the schematic diagram of a hippocampal slice (str. rad: stratum radiatum; DG: dentate gyrus; str. pyr: stratum pyramidale). **(B)** The input-output curves for the Klotho (red) and Vehicle (blue) groups show the changes in fEPSP slopes as a function of stimulation intensity. Klotho overexpression mediated a significant increase in fEPSP slope per unit of stimulation intensity (Klotho: n = 6; Vehicle: n = 5; Mann-Whitney test: ^∗∗^P < 0.05). The insets show representative fEPSP traces. Scale bar: 1 mV/5 ms. **(C)** Paired-pulse ratio (PP ratio) of fEPSPs after 2 months of Klotho (n = 9) or GFP-only (n = 8) overexpression at a stimulation strength that evoked fEPSPs at 30% of the maximal fEPSP slope. **(D)** The graph summarizes the PP ratios for the two groups utilizing fEPSPs at 45% of the maximal fEPSP slope. The insets show overlaid traces of fEPSP pairs with different inter-stimulus intervals. Scale bar: 1 mV/20 ms. **(E)** LTP induction and fEPSP potentiation were monitored for 180 min following tetanization (n = 5, Klotho: red circles; n = 4, Vehicle: blue circles). The LTP of Klotho mice showed a significant effect from the 5th to the 147th minute. Baseline fEPSP recordings did not show differences between the two groups (Klotho: red triangles; Vehicle: blue triangles). **(F)** The LTD of the fEPSP was monitored for 180 min following 900 stimuli at 1 Hz. The degree of LTD and baseline stability did not differ between the two groups (n = 4 each). The insets show representative fEPSPs for Klotho and Vehicle mice. Scale bar: 1 mV/5 ms. Brackets and asterisks indicate significant differences with P < 0.05 (Mann-Whitney test). ns: non-significant.

To determine if Klotho overexpression affects synaptic transmission, we tested CA1 hippocampal synaptic plasticity using acute hippocampal slices from Vehicle and Klotho group mice 2 months after infection with the virus (Figure 6A). To learn more about the relationship between the number of Schaffer collateral fibers and the efficiency of synaptic transmission, the initial slope of the evoked fEPSPs was measured as a function of stimulation intensity (input-output relationship). The data indicated that Klotho-overexpressing mice had a prominent increase in synaptic transmission per unit stimulation intensity compared with the Vehicle group mice. For example, the fEPSP slope value at 0.8 V was 0.6 ± 0.08 mV/ms for the Klotho group and 0.2 ± 0.05 mV/ms for the Vehicle group (Figure 6B). Details of the statistical comparisons of the input-output relationships of fEPSPs between the groups are presented in Supplementary Table 5.

Paired-pulse facilitation (PPF) is a form of short-term plasticity of synaptic transmission that occurs when the same population of synapses is stimulated twice within a millisecond delay. To this end, paired-pulse facilitation experiments with test fEPSPs that corresponded to either 30% (Figure 6C) or 45% (Figure 6D) of the maximal fEPSP slopes were conducted. Under both conditions, the PPF of the Klotho group (*n* = 9) differed significantly from that of the Vehicle group (*n* = 8) for most inter-stimulus intervals, except for the 30 and 150 ms intervals at 30% of the maximal fEPSP slope and the 30, 130, 250 and 290 ms intervals at 45% of the maximal fEPSP slope (Mann-Whitney: *P* < 0.05, Figure 6C,D). Overexpression of Klotho for 2 months reduced the PPF at a 40 ms inter-stimulus interval and 30% of the maximal fEPSP slope to 1.21 ± 0.014%, which was lower than that of the Vehicle group at 1.39 ± 0.083% (Mann-Whitney: *P* = 0.002, Figure 6C). Even at an inter-stimulus interval of 290 ms, the PPF was significantly lower in the Klotho group (1.05 ± 0.008%) than in the Vehicle group (1.12 ± 0.022%; Mann-Whitney: *P* = 0.016). Details of the statistical comparisons of the PPF between the groups are presented in Supplementary Table 6. The decline in PPF suggests that the release probability might be increased at hippocampal synapses in the Klotho mice (Thomson, 2000). An enhanced release probability could increase the size of the first fEPSP, which would be followed by a refractory period preventing the facilitation of the second fEPSP (Thomson, 2000). Indeed, we observed an enhancement of the fEPSP’s input-output characteristic of α-Klotho overexpressing mice (Figure 6B).

It is well documented that long-term potentiation (LTP) is a persistent strengthening of synapses in response to brief but rapidly repeated synaptic transmissions (Cohen et al., 1999). In contrast, repeated induction of synaptic transmissions over several minutes at a low frequency (1 Hz) weakens synapses. Such depression of synaptic transmission (long-term depression; LTD) can last for several hours under *in vitro* conditions. To test whether LTP was affected by Klotho overexpression, fEPSP potentiation was induced by one round of 100 stimuli at 100 Hz (Figure 6E) in acute hippocampal slices. The normalized fEPSP slopes at 20 min and 100 min were 164.3 ± 6.33% and 147.9 ± 6.05% for the Klotho group (*n* = 5) and 133.8 ± 4.19% and 125.0 ± 5.27% for the Vehicle group (*n* = 4), respectively (Figure 6E). To induce LTD, a 1 Hz stimulation for 15 min at 45% of the maximal fEPSP slope was applied. The normalized fEPSP slopes at 30 min and 100 min were 70.7 ± 2.35% and 83.3 ± 1.96% for the Klotho group (*n* = 4) and 86.3 ± 1.96% and 97.9 ± 5.49% for the Vehicle group (*n* = 4), respectively (Figure 6F). The two groups did not demonstrate significantly different LTD. Details of the statistical comparisons of LTP and LTD between the groups for different recording time points are presented in Supplementary Table 7. In summary, α-Klotho overexpression increased hippocampal synaptic transmission and altered paired-pulse facilitation of synaptic transmission, and enhanced activity-dependent synaptic plasticity.

## Discussion

We have here presented data showing that the overexpression of human secreted α-Klotho in the CA1 region improves memory performance in three different learning tasks and alters hippocampal synaptic transmission. Thus, overexpression of Klotho could be beneficial to counteract the decline in hippocampal α-Klotho expression in aging and therefore attenuate some of the age-dependent alterations in cognitive abilities. However, overexpression of Klotho might also negatively interfere with social behavior, as revealed here by the reduction in nest-building behavior.

### The Expression Pattern of α-Klotho

The expression pattern of α-Klotho was initially characterized at the mRNA level and revealed that gene expression occurs in the murine and rat kidney, brain and pituitary glands (Kuro-o et al., 1997). Klotho has been detected at the mRNA and protein levels to different degrees within most organs. The highest levels, however, have been found in the kidney, parathyroid gland, choroid plexus and sinoatrial node (Olauson et al., 2017). Regarding the brain it has been shown that significant Klotho mRNA levels are detectable in animals (Kuro-o et al., 1997). Several studies have described the expression of α-Klotho in cerebellar Purkinje cells as well as in, for example, the hypothalamus, thalamus and striatum (Fon Tacer et al., 2010; Lorenzi et al., 2010; Chen et al., 2013; Clinton et al., 2013; Brobey et al., 2015; Dubal et al., 2015). In addition, a number of studies have identified α-Klotho expression within the hippocampal formation (Teocchi et al., 2013; Dubal et al., 2014; Schafer et al., 2015). Regarding an age-dependent decline in α-Klotho, our data are in line with findings that Klotho expression is reduced during aging in mice (Duce et al., 2008; Masso et al., 2015). In the reduction in the soluble form of Klotho, at least, ADAM10 activity plays a role; thus, higher or lower ADAM10 activity could affect the detectable levels of transmembrane Klotho. Thus, the available data indicate that Klotho deficiency in the brain could contribute to a reduction in cognitive ability. Thus, we also studied whether overexpression in 11- to 12-month-old mice could alter their cognitive ability.

### Effects of Secreted Klotho Overexpression in the CA1 Region on Pluripotency, Synaptic Plasticity Relevant Signaling Pathways and Synaptic Transmission

Although cognitive function is affected by the Klotho expression levels (Nagai et al., 2003; Dubal et al., 2014), it is not well understood which form of Klotho (secreted or membrane-bound) mediates the modulation of cognitive ability. Thus, in this study, we utilized a CRE double-floxed expression system to overexpress human secreted Klotho in CA1 neurons of aged mice and to study the effect in several behavioral and cellular paradigms.

Since it has been shown previously that overexpression of full-length α-Klotho affects neurogenesis (Zhou et al., 2018), we tested whether human secreted Klotho expressed in CA1 neurons enhances pluripotency in the SGZ (Laszczyk et al., 2017). Several transcription factors have been described that can be utilized to evaluate the state of newly developed cells (Salech et al., 2017). We used the transcription factor sex determining region Y-box 2 (SOX2) as a marker protein for pluripotent cells. We found that Klotho overexpression enhanced the expression of SOX2 in the hippocampus, even though the secreted α-Klotho had been overexpressed within the CA1 region. Therefore, these data suggested that the secreted Klotho was distributed within the hippocampus. Additional studies are needed to determine whether the maturation of newly developed cells into neurons is promoted by secreted α-Klotho.

A further hint that secreted Klotho affects neuronal composition was the increased level of PSD-95. The increased level of PSD-95 might have been due to an increased number of synapses or to enlarged postsynaptic areas. However, the levels of presynaptic marker proteins, such as SNAP-25 and syntaxin, were not increased but were instead reduced. Only the expression level of Munc 18-1, which is required for vesicle priming, was not affected by the secreted α-Klotho. Since Munc 18-1 acts as a stabilizer of SNAP-25 and syntaxin complexes, decreased expression of SNAP-25 and syntaxin could alter presynaptic release probability (Thomson, 2000). SNAP-25 knock down experiments have shown that paired pulse facilitation of glutamatergic synapses is transformed into a paired pulse depression whereas evoked glutamatergic neurotransmission became enhanced (Antonucci et al., 2013). In our experiments, examination of the relationship between stimulation strength and synaptic transmission revealed that overexpression of α-Klotho enhanced the efficacy of synaptic transmission. On the other hand, the facilitation of synaptic transmission in the paired-pulse paradigm was attenuated in Klotho-overexpressing mice. Thus, secreted α-Klotho affected synaptic transmission by multiple alterations of presynaptic and postsynaptic signaling pathways. Previous publications have not described such effects on synaptic transmission, and the potentially involved signaling pathways remain to be elucidated.

Prolonged overexpression of human secreted α-Klotho altered the phosphorylation levels of PI3K and AKT without modulating their total expression levels. The PI3K and AKT pathways play multiple roles in the neuronal system and contribute to activity-dependent synaptic plasticity either through presynaptic modulation of vesicle release or postsynaptic modulation of synaptic composition (Sanna et al., 2002; Karpova et al., 2006).

The receptors or interaction partners of the secreted α-Klotho are only beginning to be documented; however, an interaction with FGF or FGF receptors could be postulated based on current knowledge of the signaling mechanism of α-Klotho. Insulin-related pathways might also be affected, as a close link between Klotho expression levels and insulin-dependent signaling pathways has been described (Havrankova et al., 1978; van Houten et al., 1979) that might play a role in the neural development and learning and memory (Gerozissis, 2008). Both insulin- and FGF-dependent pathways are closely linked to the PI3K signaling pathway. Other potential pathways that might be activated by soluble or secreted Klotho are erythropoietin receptor-GATA-1 pathways (Cheng et al., 2015) and growth hormone/insulin-like growth factor pathways (Rubinek et al., 2016).

### Nesting Behavior and Memory Performance

One of the striking observations in this study was the alteration in nest-building behavior in mice with enhanced Klotho levels. Nest construction is widespread throughout the animal kingdom and is especially common among small rodents; nests are important for heat conservation as well as for reproduction and shelter (Sieber et al., 1980; Deacon, 2006). In 1960, it was shown that the hippocampus plays a key role in nesting behavior (Kim, 1960), but due to the complexity of this behavior, the underlying signaling pathways remain elusive (Hall et al., 2015; Snyder-Mackler and Tung, 2017). However, it has been accepted that nest-building behavior can be an indicator of the health of female and male mice (Gaskill et al., 2013; Goto et al., 2015; Greenberg et al., 2016; Rhee et al., 2018). Altered nesting behaviors has been implicated e.g., in autism-like behavior of mice (Rhee et al., 2018). In addition, it remains unclear if the alteration in nest-building behavior was due to a general brain-wide effect of the secreted Klotho or if it was due to specific effects in the CA1 area.

As memory can be a measure of cognitive ability, we tested the effects of overexpression of human secreted α-Klotho on object recognition and OLM, two types of memory that are dependent on the hippocampus to different degrees (Haettig et al., 2011; Vogel-Ciernia and Wood, 2014). Our results showed that increased levels of Klotho improved memory performance in the object recognition and object location tasks. The object location task depends upon proper functioning of the hippocampus, and in our previous BDNF overexpression study, only this measure of memory performance was enhanced (Wang et al., 2017). One possible explanation might be that the localized overexpression of secreted Klotho increases the levels within the whole hippocampus or in the whole brain via the ventricular system, thus promoting the function of deeper brain areas that could contribute to ORM (Vogel-Ciernia and Wood, 2014). These data are in line with findings that body- and brain-wide overexpression of full-length mouse Klotho improves ORM in an Alzheimer’s disease-like animal model (Dubal et al., 2015). However, impairment of novel-object recognition has been observed in Klotho mutant mice at the age of 7 weeks. This impairment could be counteracted by a potent antioxidant (Nagai et al., 2003). Whereas we described effects on object recognition and OLM, other α-Klotho studies have described the modulation of memory performance in different learning paradigms. For example, transgenic mice with systemic overexpression of Klotho-VS, a lifespan-extending variant of the human klotho gene, have been found to age-independently perform better than control mice in Morris water maze and Y-maze tests (Dubal et al., 2014), two tests that evaluate spatial memory. Another study found that overexpression of secreted Klotho in response to infection of neurons by intraventricular injection with a lentivirus-based expression system significantly improved the performance of SAMP8 mice in the Y-maze task (Zhou et al., 2018). Surprisingly, peripheral administration of a recombinant α-Klotho fragment (KL1+KL2) was found to be sufficient to enhance memory performance in the Morris water maze and Y-maze in young, aging, and transgenic α-synuclein mice (Leon et al., 2017), even though this fragment does not cross the blood-brain barrier.

In addition to the already mentioned findings, we also showed that passive avoidance memory was strongly enhanced in response to the overexpression of secreted Klotho even 120 h after training. Such long-term enhancement has not been shown previously; however, the data are in line with a previous report showing improvement in passive avoidance memory by α-Klotho (Nagai et al., 2003; Dubal et al., 2015).

Our data confirm and extend the results published by Masso et al. (2017). Masso et al. (2017) studied the effect of overexpressed mouse secreted Klotho in response to the transduction of neurons after injection of AAVrh10 vectors in the cerebral ventricle or the dorsal hippocampus (Masso et al., 2017). After 6 months of Klotho overexpression they observed improvement in learning and memory performance in the T- and Morris water maze. In addition to the work of Mass et al., we show that specific Klotho overexpression in excitatory CA1 neurons of 11- to 12-month-old mice was sufficient to improve object location and objection recognition memory. The memory evaluated in a passive avoidance task was significantly enhanced, but insensitive to relearning. Masso et al. showed the shRNA-Klotho treated animals showed memory and/or learning problems. In addition, they provided evidences that Klotho overexpression is also efficient at 12- to 18-month-old mice (Masso et al., 2017).

As it can be noted in Figure 1B some cells were also transduced at the posterior parietal association areas layer 6 close to the lateral ventricle. We can not exclude the participation of these cells in the observed effects. The overexpression systems combined localized stereotaxic infection of CA1 neurons in the dorsal-intermediate hippocampus and CaMKII::Cre-floxed expression that allowed highly targeted Klotho overexpression. The posterior parietal association areas have been associated with interaction of the environment and with visual-spatial attention. The posterior parietal cortex of primates has been shown to be involved in working memory and task learning, however, the contribution of the layer 6 in these tasks remains not clear (Rawley and Constantinidis, 2009). Thus, a more sophisticated expression system that takes advantage of specific genetic markers for different brain areas (Fanselow and Dong, 2010) might help to further reduce the putative impact of out-of-target cell transduction on observations.

### Overexpression of Human Secreted α-Klotho in the CA1 Region Enhances the Induction of Activity-Dependent Synaptic Plasticity

We tested whether activity-dependent synaptic plasticity, such as LTP and long-term depression of hippocampal synaptic transmission, are regulated by α-Klotho. In response to the repeated 100 Hz train, we observed a significant increase in synaptic transmission within the first hours of fEPSP potentiation but not after 3 h. In contrast, a previous study showed that global overexpression of mouse full-length α-Klotho can age-dependently decrease synaptic plasticity in the hippocampal CA1 area in 6-month-old transgenic mice (Li et al., 2017). In this work, the overexpression of full-length α-Klotho was most likely driven by the human elongation factor 1a promoter (Kurosu et al., 2005; Li et al., 2017). However, in a different study, overexpression of α-Klotho enhanced activity-dependent synaptic plasticity in the dentate gyrus (Dubal et al., 2014). In contrast, hippocampal LTP was only weakly reduced in Klotho-knockout mice. This effect was only detectable when fEPSP potentiation was induced by a single theta burst (Park et al., 2013). In contrast, another study showed that mice with Klotho deficiency had a moderate improvement of LTP in the CA1 area (Li et al., 2017). This transgenic mouse strain had an α-Klotho deficiency due to an insertional mutation that disrupted the 5′ promoter region (Kuro-o et al., 1997). Regarding the long-term depression of synaptic transmission, overexpression of Klotho did not alter the induction or expression phase of long-term depression. In general, the effects of α-Klotho on synaptic plasticity are not consistent among publications; thus, more specific differentiation among transgenic mice strains, ages, and testing paradigms is needed. However, our data also suggest that secreted α-Klotho is capable of modulating synaptic transmission and synaptic plasticity.

In addition, Klotho that is released in the blood stream has been shown to promote the recovery from multiple pathological events. For example, secreted or soluble Klotho in the serum has been shown to reduce neuronal loss and neurodegeneration (Abraham et al., 2012), promote stem cells (Bian et al., 2015), and protects the heart from hyperglycemia-induced injury (Guo et al., 2018). In addition, the level of serum Klotho has been also utilized to predict the onset of multiple system atrophy (Guo et al., 2017). Thus, releasable Klotho present a very interesting humoral factor that could be helpful to find new means against neurodegenerative diseases.

## Conclusion

We detected α-Klotho in mouse brain regions and determined that the expression of Klotho in the hippocampus of mice decreased significantly during aging. Overexpression of human secreted Klotho in neurons of the CA1 area enhanced object recognition, object location, and passive avoidance memory performance. In addition, human secreted Klotho altered the nesting behavior of mice. On the cellular level, Klotho overexpression altered several basal parameters of synaptic transmission and enhanced the initial potentiation of Schaffer collateral CA1 synaptic transmission. These findings strongly support the hypothesis that enhanced expression of α-Klotho promotes synaptic plasticity-related signaling pathways and memory performance in aging. Our observations highlight the importance of Klotho in normal synaptic function and behavior and provide additional information on how to overcome the effects of age-dependent hippocampal α-Klotho downregulation on memory formation.

## Author Contributions

DL, DJ, FH, and TB conceived the project and designed the experiments. DL and DJ carried out the experiments and analyzed the data with ZL and YC help. DL, DJ, FH, and TB wrote the manuscript. All authors have approved the final version of the manuscript and agreed to be accountable for all aspects of the work.

## Conflict of Interest Statement

The authors declare that the research was conducted in the absence of any commercial or financial relationships that could be construed as a potential conflict of interest.
