# Supplementary figures and images for: Enhanced Expression of Secreted α-Klotho in the Hippocampus Alters Nesting Behavior and Memory Formation in Mice
Source: Front Cell Neurosci. 2019 Apr 2;13:133. doi: 10.3389/fncel.2019.00133 (PMC6454015; doi:10.3389/fncel.2019.00133)

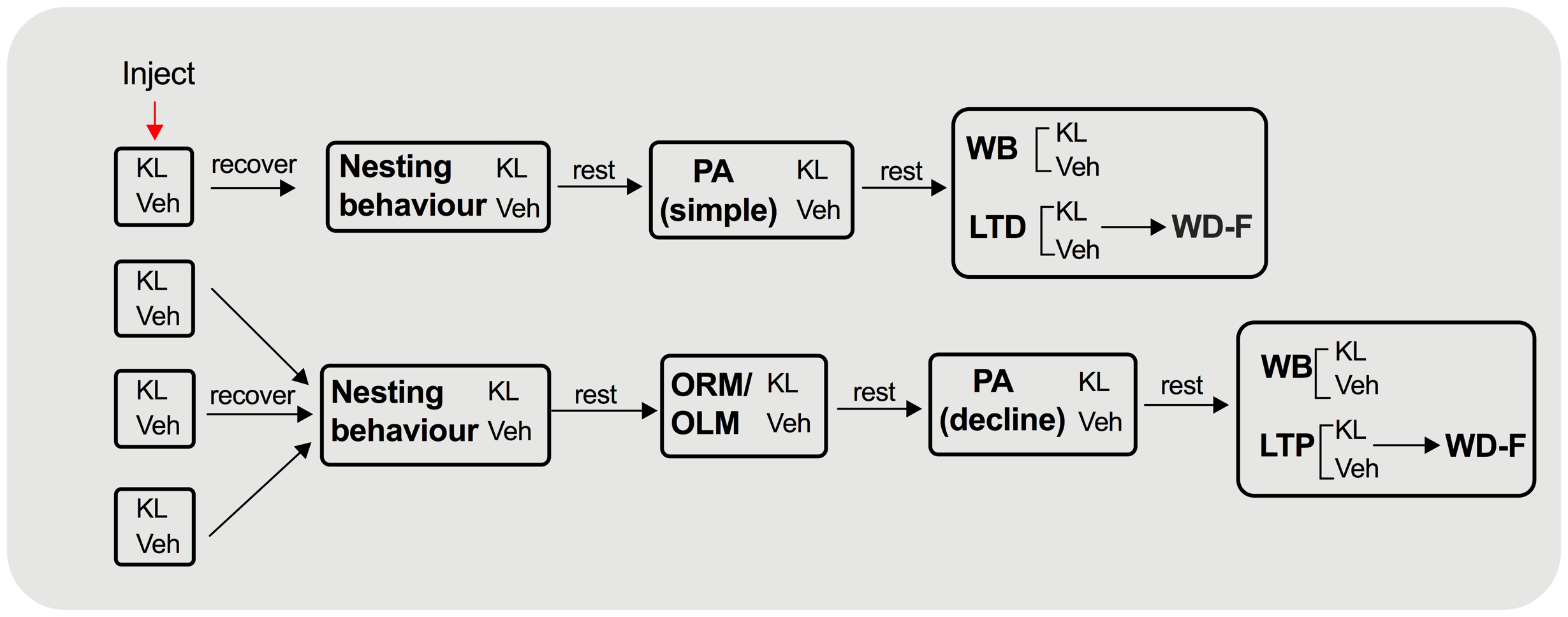

Supplement: Supplementary file 1 [file Image_1.TIFF]

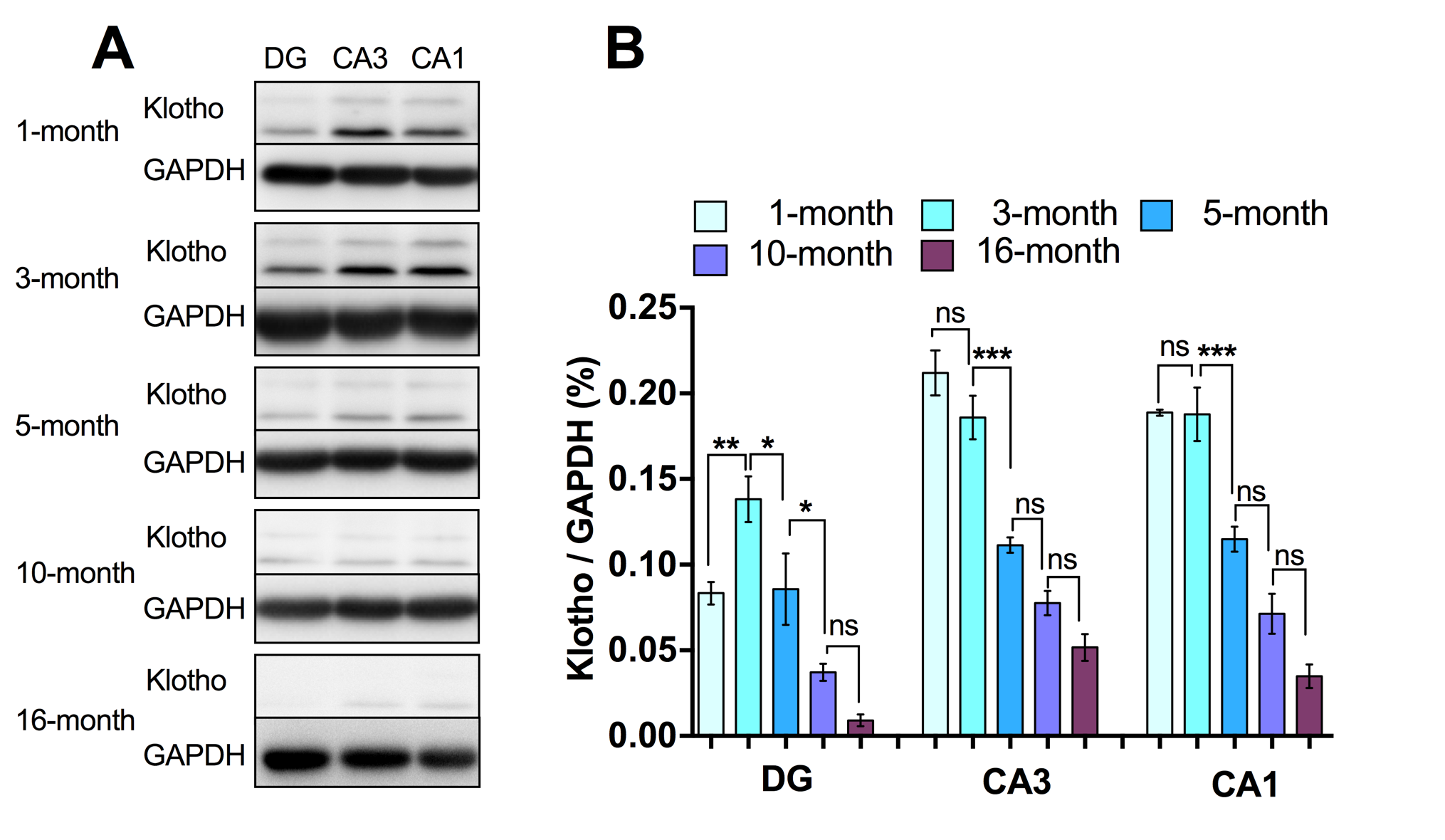

Supplement: Supplementary file 2 [file Image_2.TIFF]

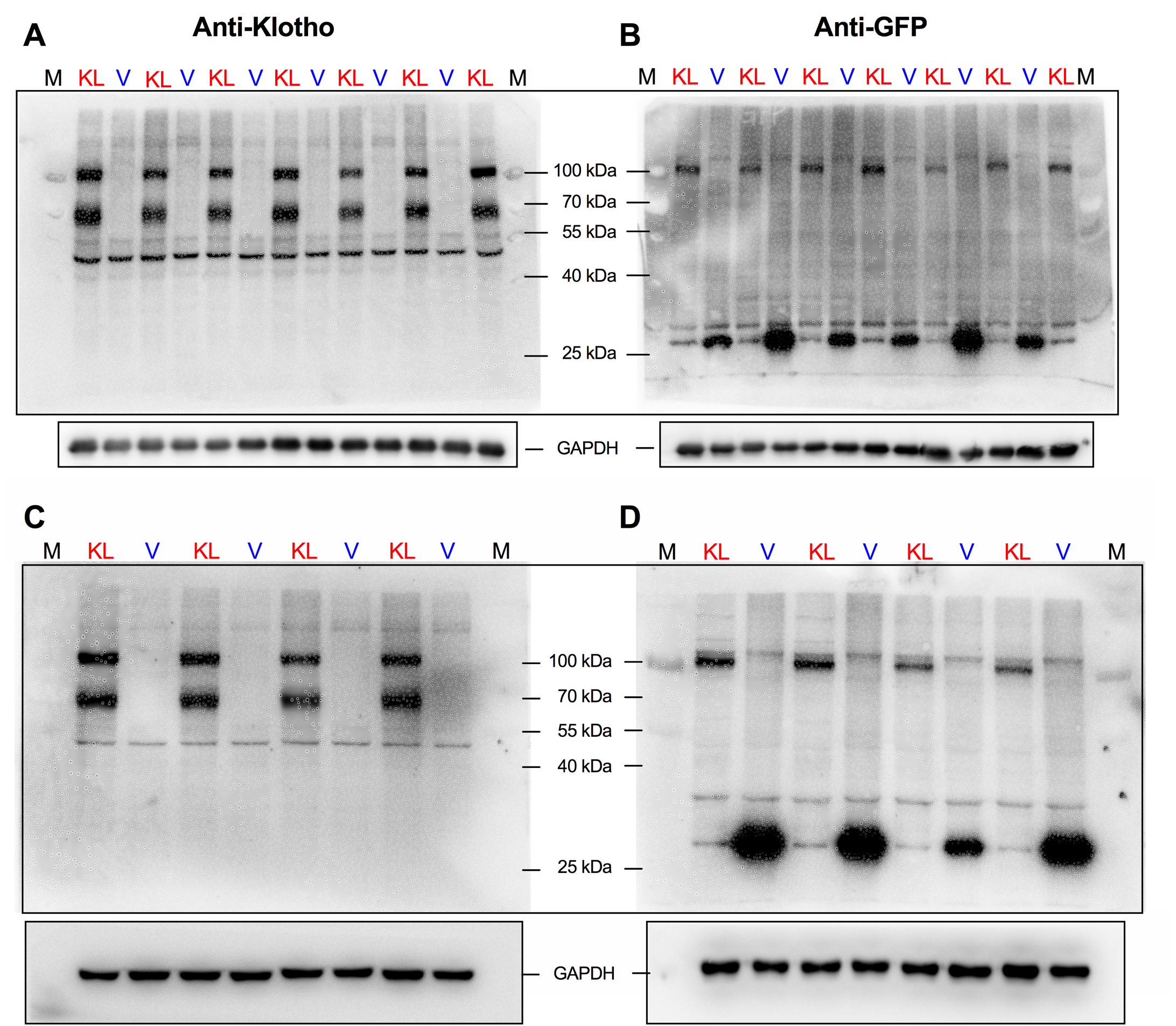

Supplement: Supplementary file 3 [file Image_3.TIFF]

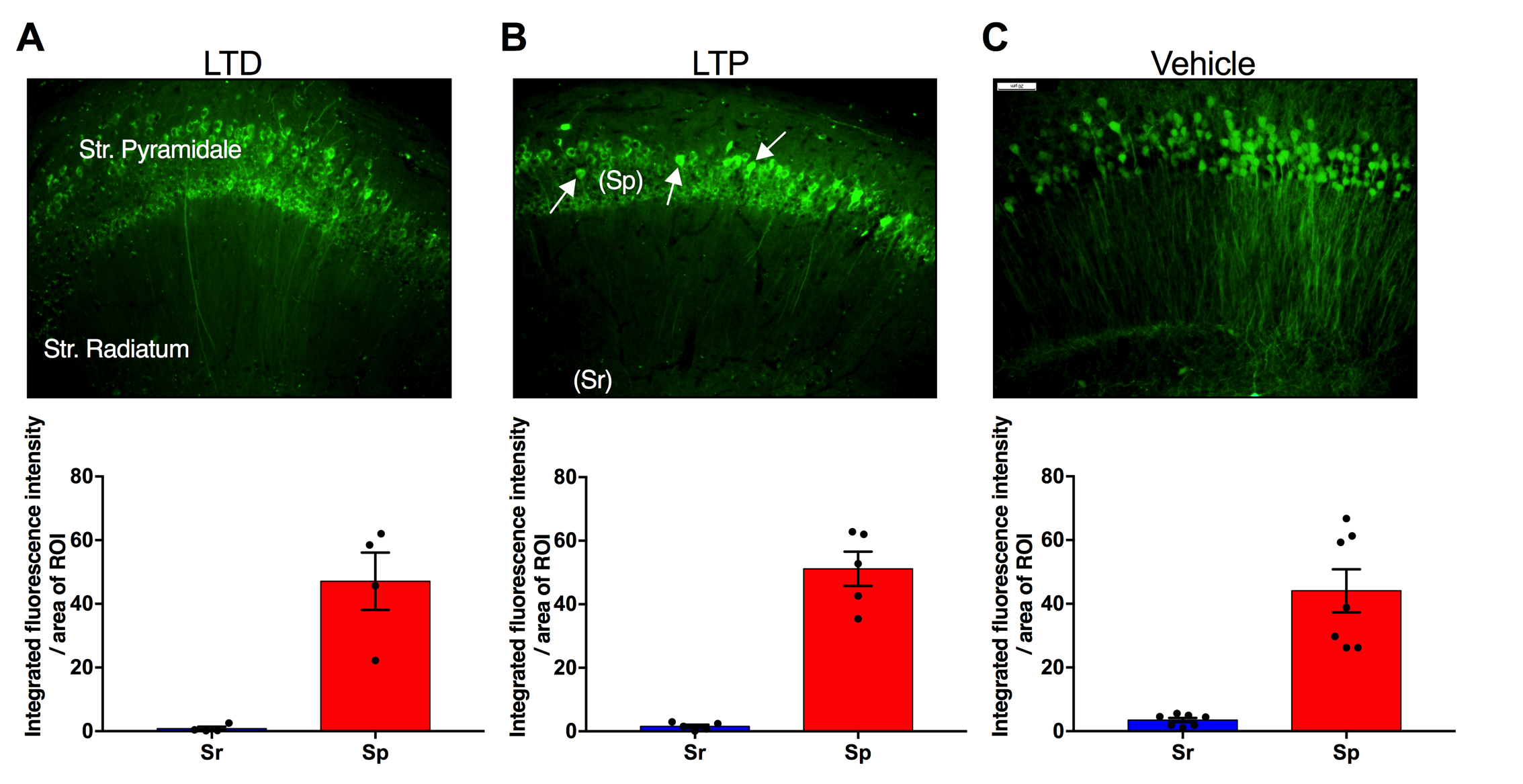

Supplement: Supplementary file 4 [file Image_4.TIFF]
