# Supplementary material for: Enhanced Expression of Secreted α-Klotho in the Hippocampus Alters Nesting Behavior and Memory Formation in Mice
Source: Front Cell Neurosci. 2019 Apr 2;13:133. doi: 10.3389/fncel.2019.00133 (PMC6454015; doi:10.3389/fncel.2019.00133)
Supplement: Supplementary file 5 [file Table_1.DOCX]

**Supplementary figures and tables**

**Supplementary Figure 1. Outline of the experimental sequence.**

Over the course of data collection several batches of mice were analyzed. One batch includes mice that were infected at the same day by either the Vehicle (Veh: GFP-only) or secreted-alpha-Klotho (KL) AAV9s. After recovery of the mice the different tasks were conducted as outlined in the graph. After the behaviour experiments the hippocampi were collected for protein biochemical evaluation. In addition, the hippocampi of some of the animals were further analyzed by electrophysiology and wide-field fluorescence (WD-F) microscopy. PA: Passive-avoidance; ORM: Object recognition memory; OLM: Object location memory; LTD: Long-term depression; LTP: Long-term potentiation.

**Supplementary Figure 2. Age-dependent regulation of endogenous secreted alpha-Klotho expression in the hippocampal formation.**

**(A)** Representative Western blots of anti-Klotho and corresponding GAPDH are presented at the left panel for DG, CA3 and CA1 at different age of the mice. **(B)** Normalized alpha-Klotho values at different age and regions are summarized in the bar graph (mean ± SEM). Adjacent groups in the bar graph that differ significantly with each other are indicated with brackets and asterisks (Mann-Whitney test: *P < 0.05, **P < 0.01, ***P < 0.001, n = 3).

**Supplementary Figure 3.** **Verification of Klotho overexpression with non-attached GFP and GFP-only in hippocampal CA1 area of CaMKII::Cre mice by fluorescence microscopy.**

A) Verification of the Klotho overexpression in slices from LTD experiments. A representative fluorescence image of CA1 neurons overexpressing Klotho with non-attached GFP (green) has been shown. Neurons with Klotho overexpression are indicated by the GFP-fluorescence (green, examples: white arrow). The bar diagram below summarizes the integrated fluorescence intensity dived by the area of the region of interest (ROI) within distal part of str. radiatum (sr) and the str. pyramidale (sp). B) The representative fluorescence image of a hippocampal slice from the LTP experiments indicates the successful transduction of CA1 neurons. The bar diagram below summarizes the GFP fluorescence. C) A representative fluorescence image of a slice after transduction with a GFP-only expression vector (Vehicle) is presented. The bar diagram below the image summarized the fluorescence intensity in all control (Vehicle) slices from the LTP and LTD experiments.

**Supplementary Figure 4.** **Verification of Klotho overexpression in CaMKII::Cre mice that participated in different behavioral experiments by Western blotting.**

Transduction of hippocampal neurons was utilized with 5 different batches. Every batch consisted of 4-5 animals for each group of Klotho and GFP-only AAV9 particle stereotaxic injections. 11 animals were tested for the level of Klotho expression. The remaining animals were tested for expression level and localization by fluorescence microscopy and data are presented in Supplementary Figure 3) The level of Klotho for 7 Klotho overexpressing (KL) and 6 GFP-only (V: Vehicle) animals after finalization of all behavioral experiments are presented. The marker columns (lanes) are indicated with M. B) The corresponding blot for the GFP-immunosignal is presented. A strong GFP immunosignal is at around 100 kDa in the KL-lanes, indicating that some GFP remained attached to the Klotho protein. Around 27 kDa is the GFP-only or GFP non-attached immunosignal. C) and D) depict Western blots against Klotho or GFP for 4 other animals of Klotho (KL) and GFP-only (V) groups. The Abcam 181373 Klotho antibody had been used to test for Klotho expression.
