# Supplementary material for: Enhanced Expression of Secreted α-Klotho in the Hippocampus Alters Nesting Behavior and Memory Formation in Mice
Source: Front Cell Neurosci. 2019 Apr 2;13:133. doi: 10.3389/fncel.2019.00133 (PMC6454015; doi:10.3389/fncel.2019.00133)
Supplement: Supplementary file 6 [file Table_2.DOCX]

**Supplementary tables**

**Supplementary Table 1. Statistical comparison of Western Blot data**

| **Fig 1&2 WB** | ***: non-normal distribution**  **(p<0.05, Shapiro-Wilk Test)** | | | | **t-test/ #:Mann-Whitney** | | | |
| --- | --- | --- | --- | --- | --- | --- | --- | --- |
|  |  |  | **Equal variance assumed** | |  |  | |  |
|  | **Veh mean±SEM** | **Klotho mean±SEM** | **F value** | **p value for F** | **t value** | **df** | **p values** | |
| **PSD-95** | 5.486±0.1762 | 6.505±0.3399 | 1.397 | 0.282 | -2.661 | 6 | 0.037 | |
| **SOX2** | 0.013±0.0026 | 0.028±0.0043 | 2.925 | 0.118 | -3.067 | 11 | 0.011 | |
| **PI3K** | 0.050±0.0041 | 0.051±0.0038 | 0.189 | 0.672 | -0.055 | 11 | 0.957 | |
| **p-PI3K** | 0.018±0.0015 | 0.025±0.0016 | 0.562 | 0.469 | -3.269 | 11 | 0.007 | |
| **Akt*** | 0.634±0.0514 | 0.517±0.0300 | 1.999 | 0.185 | 2.033 | 11 | 0.067/#0.25 | |
| **p-Akt** | 0.163±0.0220 | 0.316±0.0535 | 4.980 | 0.050 | -2.653 | 11 | 0.022 | |
| **SNAP25** | 0.993±0.1046 | 0.471±0.0652 | 0.060 | 0.811 | 4.372 | 11 | 0.001 | |
| **Munc 18-1** | 0.653±0.0282 | 0.640±0.0278 | 0.011 | 0.918 | -0.336 | 11 | 0.743 | |
| **Syntaxin 1A** | 0.531±0.0489 | 0.313±0.0436 | 0.018 | 0.896 | 3.330 | 11 | 0.007 | |

**Supplementary Table 2. Statistical comparison of Nesting behaviour from 4 batches (different stereotaxic injection days). Every batch consisted of 4 animals for each group.**

| **Fig 3 Nesting Behaviour** | |  | **Mann-Whitney Test** | |
| --- | --- | --- | --- | --- |
|  | N= 4 batches | N= 4 batches |  |  |
|  | unit: number of mesh cells with cotton balls / total number of mesh cells (15) | | |  |
| **Fig 3B** | **Veh mean±SEM** | **Klotho mean±SEM** | **Z value** | **p value for Z** |
| **0 h** | 0.200±0.0000 | 0.200±0.0000 | 0.000 | 1.000 |
| **0.5 h** | 0.383±0.0167 | 0.767±0.0430 | -2.366 | 0.018 |
| **1 h** | 0.367±0.0193 | 0.800±0.0385 | -2.366 | 0.018 |
| **2 h** | 0.350±0.0167 | 0.800±0.0471 | -2.381 | 0.017 |
| **3 h** | 0.333±0.0000 | 0.683±0.0167 | -2.530 | 0.011 |
| **4 h** | 0.333±0.0000 | 0.633±0.0193 | -2.494 | 0.013 |
| **11 h** | 0.267±0.0000 | 0.550±0.0167 | -2.530 | 0.011 |
| **24 h** | 0.267±0.0000 | 0.266±0.0000 | 0.000 | 1.000 |
|  |  |  |  |  |
|  | unit: Intact cotton balls / total cotton balls (are 30) | |  |  |
| **Fig 3C** | **Veh mean±SEM** | **Klotho mean±SEM** | **Z value** | **p value for Z** |
| **0 h** | 1.000±0.0000 | 1.000±0.0000 | -1.000 | 0.317 |
| **0.5 h** | 0.458±0.0285 | 0.892±0.0083 | -2.323 | 0.020 |
| **1 h** | 0.217±0.0289 | 0.850±0.0096 | -2.337 | 0.019 |
| **2 h** | 0.167±0.0136 | 0.825±0.0083 | -2.337 | 0.019 |
| **3 h** | 0.067±0.0136 | 0.808±0.0083 | -2.337 | 0.019 |
| **4 h** | 0.042±0.0083 | 0.783±0.0167 | -2.428 | 0.015 |
| **11 h** | 0.017±0.0096 | 0.758±0.0160 | -2.337 | 0.019 |
| **24 h** | 0.000±0.0000 | 0.700±0.0136 | -2.477 | 0.013 |

**Supplementary Table 3. Statistical comparisons of Passive Avoidance data**

| **Fig 4 Passive Avoidance** | |  | **Mann-Whitney Test** | |
| --- | --- | --- | --- | --- |
|  | unit: in sec | unit: in sec |  |  |
| **Fig 4C** | n=8 | n=8 |  |  |
|  | **Veh mean±SEM** | **Klotho mean±SEM** | **Z value** | **p value for Z** |
| **0 h (Training)** | 11.234±2.0255 | 12.795±3.7692 | -0.105 | 0.916 |
| **24 h (Test)** | 51.783±12.4098 | 135.290±15.1717 | -3.046 | 0.002 |
|  |  |  |  |  |
| **Fig 4D** | n=9 | n=14 |  |  |
|  | **Veh mean±SEM** | **Klotho mean±SEM** | **Z value** | **p value for Z** |
| **0 h (Training)** | 15.604±2.4516 | 14.864±2.6354 | -0.504 | 0.614 |
| **1 h (Training)** | 75.314±14.8986 | 49.989±13.2028 | -1.449 | 0.147 |
| **24 h (Test)** | 210.353±37.7456 | 227.936±28.4436 | -0.394 | 0.694 |
| **48 h (Test)** | 162.488±24.4276 | 153.903±32.7919 | -0.063 | 0.950 |
| **72 h (Test)** | 182.782±38.2466 | 183.564±29.6609 | -0.286 | 0.775 |
| **96 h (Test)** | 74.222±32.4214 | 184.810±28.8314 | -2.396 | 0.017 |
| **120 h (Test)** | 60.504±24.8419 | 156.861±30.2760 | -2.081 | 0.037 |

**Supplementary Table 4. Statistical comparisons of data from ORM and OLM**

| **Fig 5 Object Recognition Memory (ORM) and Object Location Memory (OLM)** | | | | |
| --- | --- | --- | --- | --- |
|  |  |  |  |  |
|  | unit: DI in % | unit: DI in % | **Mann-Whitney Test** | |
| F**ig 5B ORM** | n=9 | n=14 |  |  |
|  | **Veh mean±SEM** | **Klotho mean±SEM** | **Z value** | **p value for Z** |
| Training | 1.044±1.1813 | -0.454±0.7812 | -0.756 | 0.450 |
| Test | 1.229±1.8258 | 37.157±2.8337 | -3.969 | <0.001 |
| after 5 days rest | |  |  |  |
| F**ig 5C OLM** |  |  |  |  |
|  | **Veh mean±SEM** | **Klotho mean±SEM** | **Z value** | **p value for Z** |
| Training | -0.476±1.6172 | -2.330±2.0334 | -0.315 | 0.753 |
| Test | 1.412±4.5557 | 32.403±2.8783 | -3.654 | <0.001 |

**Supplementary Table 5. Statistical comparisons of Input - Output characteristic of fEPSPs**

| **Fig 6 Input - Output characteristic of fEPSPs** | | |  |  |
| --- | --- | --- | --- | --- |
|  | unit: slope (mV/ms) | unit: slope (mV/ms) | **Mann-Whitney Test** | |
|  | n=5 | n=6 |  |  |
| **Fig 6B IO** | **Veh mean±SEM** | **Klotho mean±SEM** | **Z value** | **p value for Z** |
| 0.0 V | 0.068±0.0152 | 0.053±0.0208 | -0.548 | 0.584 |
| 0.2 V | 0.061±0.0214 | 0.013±0.0107 | -1.775 | 0.076 |
| 0.4 V | 0.071±0.0149 | 0.125±0.0409 | -1.095 | 0.273 |
| 0.6 V | 0.103±0.0323 | 0.381±0.0641 | -2.739 | 0.006 |
| 0.8 V | 0.192±0.0485 | 0.611±0.0851 | -2.739 | 0.006 |
| 1.2 V | 0.394±0.0916 | 1.031±0.1011 | -2.556 | 0.011 |
| 1.6 V | 0.621±0.1371 | 1.400±0.1257 | -2.373 | 0.018 |
| 2.0 V | 0.807±0.1634 | 1.740±0.1510 | -2.373 | 0.018 |
| 2.4 V | 0.963±0.1904 | 2.000±0.1916 | -2.373 | 0.018 |
| 2.8 V | 1.100±0.1965 | 2.208±0.2343 | -2.191 | 0.028 |
| 3.4 V | 1.224±0.2084 | 2.406±0.2707 | -2.373 | 0.018 |
| 4.0 V | 1.316±0.1927 | 2.491±0.3057 | -2.556 | 0.011 |
| 5.0 V | 1.505±0.2048 | 2.461±0.2722 | -2.373 | 0.018 |

**Supplementary Table 6. Statistical comparisons of Paired Pulse Facilitation**

| **Fig 6C PPF at 30% of fEPSP maximum** | | | **Mann-Whitney Test** | |
| --- | --- | --- | --- | --- |
|  | unit: P2/P1  n=8 | unit: P2/P1  n=9 |  |  |
|  | **Veh mean±SEM** | **Klotho mean±SEM** | **Z value** | **p value for Z** |
| 20 ms | 1.329±0.0782 | 1.238±0.0150 | -0.481 | 0.630 |
| 40 ms | 1.385±0.0827 | 1.205±0.0142 | -3.079 | 0.002 |
| 50 ms | 1.410±0.0817 | 1.211±0.0127 | -3.464 | 0.001 |
| 70 ms | 1.392±0.0800 | 1.197±0.0154 | -3.175 | 0.001 |
| 90 ms | 1.344±0.0631 | 1.180±0.0128 | -2.598 | 0.009 |
| 110 ms | 1.345±0.0765 | 1.170±0.0143 | -2.502 | 0.012 |
| 130 ms | 1.301±0.0587 | 1.153±0.0135 | -2.983 | 0.003 |
| 150 ms | 1.246±0.0471 | 1.144±0.0165 | -1.828 | 0.068 |
| 170 ms | 1.228±0.0517 | 1.122±0.0094 | -2.502 | 0.012 |
| 190 ms | 1.198±0.0358 | 1.094±0.0112 | -2.791 | 0.005 |
| 210 ms | 1.176±0.0327 | 1.086±0.0113 | -2.694 | 0.007 |
| 230 ms | 1.157±0.0359 | 1.068±0.0121 | -2.117 | 0.034 |
| 250 ms | 1.138±0.0304 | 1.059±0.0089 | -2.791 | 0.005 |
| 270 ms | 1.134±0.0194 | 1.043±0.0090 | -3.175 | 0.001 |
| 290 ms | 1.119±0.0222 | 1.049±0.0084 | -2.406 | 0.016 |
|  |  |  |  |  |
| **Fig 6D PPF at 45% of fEPSP maximum** | | | **Mann-Whitney Test** | |
|  | n=8 | n=9 |  |  |
|  | **Veh mean±SEM** | **Klotho mean±SEM** | **Z value** | **p value for Z** |
| 20 ms | 1.250±0.0587 | 1.212±0.0186 | -0.267 | 0.790 |
| 40 ms | 1.319±0.0788 | 1.182±0.0095 | -3.021 | 0.003 |
| 50 ms | 1.344±0.0778 | 1.184±0.0094 | -3.199 | 0.001 |
| 70 ms | 1.338±0.0768 | 1.178±0.0109 | -3.199 | 0.001 |
| 90 ms | 1.303±0.0632 | 1.164±0.0097 | -3.199 | 0.001 |
| 110 ms | 1.276±0.0548 | 1.163±0.0099 | -2.577 | 0.010 |
| **130 ms** | **1.251±0.0550** | **1.152±0.0135** | **-1.777** | **0.076** |
| 150 ms | 1.241±0.0482 | 1.138±0.0108 | -2.488 | 0.013 |
| 170 ms | 1.211±0.0448 | 1.129±0.0119 | -1.955 | 0.051 |
| 190 ms | 1.180±0.0403 | 1.096±0.0108 | -2.310 | 0.021 |
| 210 ms | 1.163±0.0384 | 1.086±0.0091 | -2.577 | 0.010 |
| 230 ms | 1.150±0.0341 | 1.074±0.0110 | -2.310 | 0.021 |
| **250 ms** | **1.131±0.0340** | **1.066±0.0093** | **-1.866** | **0.062** |
| 270 ms | 1.124±0.0357 | 1.036±0.0094 | -2.843 | 0.004 |
| **290 ms** | **1.100±0.0327** | **1.046±0.0088** | **-1.688** | **0.091** |

**Supplementary Table 7. Statistical comparisons of LTP and LTD**

| **Fig 6E LTP** |  |  | **Mann-Whitney Test** | |
| --- | --- | --- | --- | --- |
|  | unit: slope (mV/ms) in % to baseline  n=4 | unit: slope (mV/ms) in % to baseline  n=5 |  |  |
|  | **Veh mean±SEM** | **Klotho mean±SEM** | **Z value** | **p value for Z** |
| -20 min | 101.851±1.8481 | 99.026±1.3709 | -1.225 | 0.221 |
| 20 min | 133.753±4.1902 | 164.278±6.3311 | -2.449 | 0.014 |
| 60 min | 129.957±5.9950 | 153.202±5.1956 | -2.205 | 0.027 |
| 100 min | 125.007±5.2673 | 147.888±6.0499 | -2.205 | 0.027 |
| 140 min | 125.654±4.1225 | 145.689±4.5923 | -2.449 | 0.014 |
| 180 min | 123.271±7.6923 | 139.988±4.3392 | -1.470 | 0.142 |
| **Fig 6F LTD** |  |  |  |  |
|  | n=4 | n=4 |  |  |
|  | **Veh mean±SEM** | **Klotho mean±SEM** | **Z value** | **p value for Z** |
| -20 min | 98.082±1.2565 | 96.848 ±3.9279 | -0.218 | 0.827 |
| 20 min | 61.079±3.1551 | 46.119±5.6544 | -1.528 | 0.127 |
| 60 min | 99.600±9.4243 | 87.342±5.8932 | -1.091 | 0.275 |
| 100 min | 97.910 ±5.4882 | 83.343±1.9646 | -1.964 | 0.050 |
| 140 min | 99.061±2.1410 | 89.290±0.1074 | -1.732 | 0.083 |
| 180 min | 94.974±5.5084 | 93.388±4.4861 | -0.577 | 0.564 |
|  |  |  |  |  |
